# Multi-centre phase IV trial to investigate the immunogenicity of a new liquid formulation of recombinant human growth hormone in adults with growth hormone deficiency

**DOI:** 10.1007/s40618-017-0818-4

**Published:** 2018-02-27

**Authors:** G. Johannsson, K. Nespithal, U. Plöckinger, V. Alam, M. McLean

**Affiliations:** 1 Department of Endocrinology, Institute of Medicine, Sahlgrenska Academy, University of Gothenburg, Medicinaregatan 3, 41 315 Gothenburg, Sweden; 2 Merck KGaA, Darmstadt, Germany; 3 Interdisciplinary Centre of Metabolism: Endocrinology, Diabetes and Metabolism, Campus Virchow-Klinikum, Charité-Universitätsmedizin Berlin, Berlin, Germany; 4 Global Clinical Development Centre, EMD Serono Research and Development Institute, Inc. (a business of Merck KGaA, Darmstadt, Germany), Billerica, MA USA; 5 School of Medicine, University of Western Sydney, Sydney, Australia

**Keywords:** Recombinant human growth hormone, Binding antibodies, Neutralising antibodies, Liquid formulation, Adult growth hormone deficiency

## Abstract

**Purpose:**

To investigate whether a new liquid formulation of recombinant human growth hormone (r-hGH) induces the production of binding antibodies (BAbs) in adults with congenital or adult-onset growth hormone deficiency (GHD).

**Methods:**

Men or women aged 19–65 years with adult growth hormone deficiency who were r-hGH-naïve or had stopped treatment ≥ 1 month before screening were treated with between 0.15 and 0.30 mg/day r-hGH liquid formulation for 39 weeks. The primary endpoint was the proportion of patients who developed BAbs at any time. Secondary endpoints were the proportion of patients with BAbs who became positive for neutralising antibodies, the effects on biomarkers of r-hGH exposure, safety, and adherence to treatment downloaded from the easypod™ connect software.

**Results:**

Seventy-eight patients (61.5% men) with mean age 44.5 years (range 21–65) started and 68 (87.2%) completed the 39-week treatment period. 82.1% were treatment naïve; all were negative for BAbs to r-hGH at baseline. The median (interquartile range) duration of treatment [273 (267.0–277.0) days] was consistent with patients receiving the required doses, and mean treatment adherence measured using easypod™ connect was 89.3%. The proportion of patients who developed BAbs was 0% (95% confidence interval 0–4.68%) and biomarker profiles were consistent with exposure to r-hGH. 92.3% of patients reported ≥ 1 adverse event during treatment. Most events were mild or moderate and no new safety concerns were detected.

**Conclusions:**

The low immunogenicity profile of the liquid formulation was consistent with that for the freeze-dried formulation, and no new safety concerns were reported.

## Introduction

Growth hormone (GH) replacement therapy is indicated for the treatment of short stature due to growth hormone deficiency (GHD) in adults and children [1]. It has been available to treat children with GHD since the 1960s, with GH initially obtained from pituitary extracts [2] but later replaced by recombinant formulations of human GH (r-hGH), which have been available since the 1980s [3, 4]. The treatment goal is to increase linear growth and reach target height in children [5]. In addition, GH replacement therapy has been shown to have beneficial effects in adults with GHD, particularly on body composition, bone metabolism, cardiac function and quality of life [6–12].

The long-term use of recombinant therapeutic proteins, including r-hGH, needs to be associated with a favourable safety profile with regard to immunogenicity. This includes the absence of immune-related reactions, such as anaphylaxis, and the development of antibodies to the therapeutic protein, which may affect not only the efficacy of the therapeutic protein, but also lead to cross-reactivity with endogenous proteins [12]. Antibodies that bind to epitopes not linked to the activity of the therapeutic protein (binding antibodies [BAbs]) may affect the pharmacokinetics of the protein but are associated with few direct clinical consequences [13]. Antibodies that interfere with the biological activity (neutralising antibodies [NAbs]), by binding at or near the active site or by causing conformational changes, can lead to decreased efficacy [13].

Saizen^®^ (r-hGH, Merck KGaA, Darmstadt, Germany) is a formulation of r-hGH that is marketed for the long-term treatment of children and adolescents with growth failure due to inadequate secretion of endogenous GH, Turner syndrome, renal failure, and growth disturbance in short children born small for gestational age (SGA). In adults, Saizen^®^ is indicated for GHD of childhood or adult (AGHD) onset [14].

Historical assessments of the immunogenicity of the currently available freeze-dried formulation of Saizen^®^ and other r-hGH formulations report an immunogenic potential < 10% [15–17]. A new liquid formulation of Saizen^®^ for injection has demonstrated bioequivalence to the freeze-dried formulation of Saizen^®^ [18]; however, the immunogenicity of the new formulation has not yet been studied.

The primary aim of this study was to determine whether Saizen^®^ solution for injection induces BAbs in patients with AGHD at any time during a 39-week treatment course. The secondary aims were to determine the proportion of patients with BAbs who also developed NAbs, to assess the overall pharmacodynamic profile in patients with or without antibodies, to assess the safety of Saizen^®^ solution for injection and to assess adherence to the liquid formulation based on data recorded using the easypod™ electronic injector device [19].

## Methods

### Study design and outcomes

This open-label, single-arm, 39-week, phase IV trial was conducted in patients with AGHD in 20 centres in five countries (Australia, Czech Republic, Germany, Sweden and UK).

The primary outcome of the study was to determine the proportion of patients who developed BAbs to Saizen^®^ at any time during the 39-week trial. The secondary outcomes were the proportion of patients with BAbs who became positive for NAbs; the effects of Saizen^®^ on the GH biomarkers insulin-like growth factor 1 (IGF-I), insulin-like growth factor binding protein 3 (IGFBP-3) and IGF-I SDS; adherence to treatment based on data downloaded from the easypod™ electronic injection device (proportion of injections received vs. injections prescribed; 80% was defined as the acceptable level of adherence); and safety endpoints.

### Study registration and approval

At each study centre, the protocol and informed consent form for the study were reviewed and approved by a duly constituted institutional review board or independent ethics committee. The trial was explained to all patients by the investigator. Informed consent was obtained from all individual participants included in the study.

All procedures performed in this study involving human participants were approved by the institutional and/or national research ethics committee and complied with the 1964 Helsinki declaration and its later amendments or comparable ethical standards. The study is registered with clinicaltrials.gov (NCT01806298).

### Patients

Men or women with documented AGHD who were r-hGH naïve or whose previous r-hGH treatment had stopped ≥ 1 month before enrolment, were negative for BAbs at screening and had a body mass index ≤ 40 kg/m^2^ were eligible. Patients with a history of anti-r-hGH antibodies or with other health conditions were excluded.

### Protocol

Following enrolment, patients had a 6-week screening period followed by daily subcutaneous injections of Saizen^®^ r-hGH for 39 weeks/9 months via the easypod™ electronic injection device and a 2-week off-treatment follow-up period. Saizen^®^ was provided in multidose 6 mL cartridges at a concentration of 5.83 mg/mL. The dosing regimen complied with local product labelling, with a starting dose of 0.2 mg/day in men and 0.3 mg/day in women and 0.1–0.2 mg/day in older (> 60 years) individuals (lowest starting dose was 0.15 mg/day). Doses could be increased according to biochemical and clinical responses. The first injection was given by qualified clinic staff at the baseline visit and, thereafter, daily injections were administered by the participant, preferably at bedtime. Patients attended eight scheduled visits [screening, day 1 (baseline), and weeks 2, 8, 16, 29, 39 and 41] over a maximum of 47 weeks.

### Assays

#### BAbs assay

The presence of BAbs was investigated using a three-tiered approach. The screening assay comprised an electroluminescence (ECL) format. Samples, blanks and controls [sheep polyclonal anti-r-hGH (Biocheck Inc., Foster City, CA, USA)] were diluted 1:10 with assay buffer plus a biotin–Saizen conjugate and a Ruthenium–Saizen^®^ conjugate (both from MicroCoat Biotechnologie GmbH, Germany) and incubated at 25 °C for 1 h.

Samples were then transferred to a 96-well high-bind streptavidin plate and incubated for a further hour at 25 °C. The blocked streptavidin plate was washed three times with PBS-Tween. Read buffer was added to each well, and the relative light unit signals were read using a MSD 2400 imager (MSD, Rockville, MA, USA). Samples that were putatively positive for antibodies against Saizen^®^ were identified using a statistically determined cutoff point with the aim of having a 5% false-positive rate.

Putative positive samples underwent further testing in a confirmatory assay, in which samples were tested blank and in the presence of Saizen^®^. If the percentage inhibition in the presence of Saizen^®^ was above the specificity cutoff point, the sample was categorised as a true positive. Quasi-quantification by titration was performed on all true positive samples; results were expressed as the log of the dilution factor for the last positive sample. The assay was fully validated according to the current regulatory guidelines. The assay sensitivity was 22.3 µg/mL for the positive control and the inter-assay precision [expressed as the coefficient of variation (%)] of the screening assay did not exceed 7.9%. In the confirmatory assay, the inter-assay precision of the positive controls was < 3.1%. Specificity of the positive control antibody was demonstrated by the absence of immune depletion with an excess of prolactin. Drug tolerance (defined as the ability to detect the low and the high positive controls as positive in the assay in the presence of the stated amount of drug in a sample) was 400 ng/mL for the low positive control and 1600 ng/mL for the high positive control.

#### NAbs assay

A competitive luminescence bioassay based on the functional reconstruction and activation of the GH receptor in U2OS GHR-JAK2 cells was planned for the assay of NAbs. The assay was fully validated according to the current regulatory guidelines. The theoretical sensitivity of the assay was calculated using the raw responses as 386 mg/mL (equivalent to 4.8 µg/mL in 100% serum) of the positive control. The inter-assay precision of the screening assay controls did not exceed 18.8%. In the confirmatory assay, the inter-assay precision of the positive controls was < 12.3%. The highest concentration of r-hGH tolerated (defined as the ability to detect the low and the high positive controls as positive in the assay in the presence of the stated amount of drug in a sample) was 15.6 ng/mL in 8% serum for the low positive control (equivalent to 195 ng/mL in 100% serum) and 62.5 ng/mL in 8% serum for the high positive control (equivalent to 781 mg/mL in 100% serum).

#### Biomarker assays

IGF-I and IGFBP-3 assays were performed at a designated central laboratory (Europins Global Central Laboratory, Netherlands), in accordance with all applicable quality standards (https://www.eurofins.com/contact-us/worldwide-interactive-map/the-netherlands/eurofins-central-laboratory/).

### Statistical analysis

The expected proportion with a positive response for BAbs was 3%, based on a report in the literature [17]. Based on this estimate, to enable screening for antibody formation in the study reported here, a target enrolment was set for 77 patients, and data from 70 participants who completed the trial were required to observe at least one event with an actual incidence of 3% at a probability of 90.4%.

**Fig. 1:**
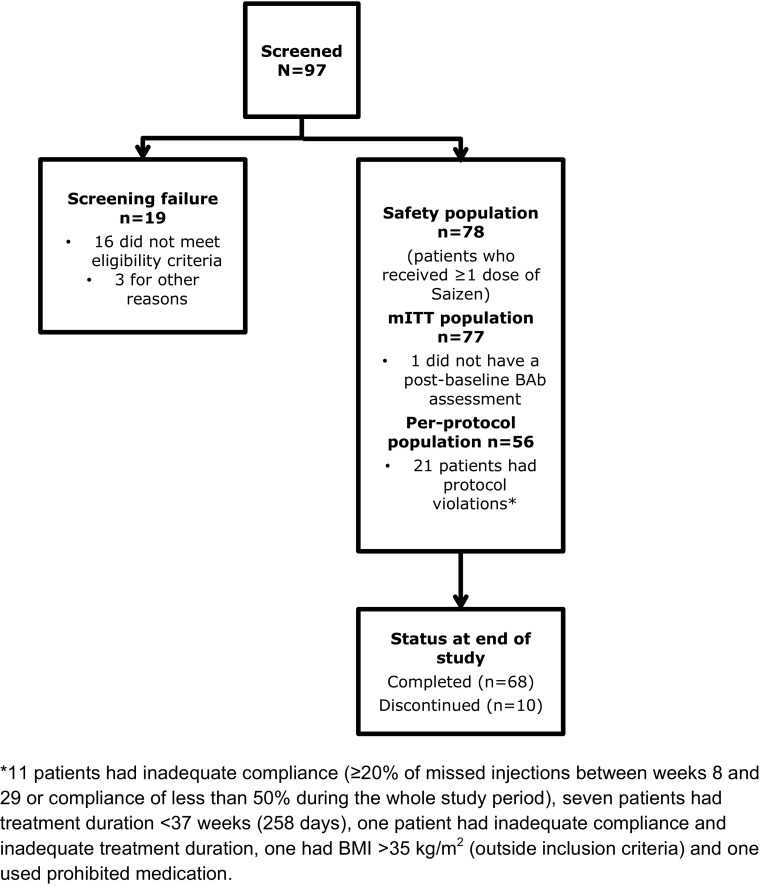
Patient flow throughout the study. *mITT* modified intention to treat, *BAb* binding antibodies, *BMI* body mass index

The primary outcome was measured in the modified intention-to-treat (mITT) population, which comprised all participants who received at least one dose of Saizen^®^ and had at least one post-baseline assessment for BAbs (Fig. 1).

The secondary outcomes were measured in the safety population, which comprised all participants who received at least one dose of Saizen^®^ (Fig. 1). Secondary endpoints were analysed using descriptive summaries on available data only and no missing data imputation procedure was used in this study.

## Results

### Patient demographics

Ninety-seven patients were screened, of whom 19 were considered screen failures (Fig. 1): no patients failed screening because of the presence of BAbs. 78 patients received at least one dose of Saizen^®^ (safety population), 77 had at least one post-baseline BAbs assessment (mITT population) and 56 (73% of mITT population) completed the trial without major protocol deviations (per-protocol population). Patient demographics are shown in Table 1. The classifications for endocrine disorders are presented exactly as entered by the treating physicians. The 22 cases with FSH deficiency and LH deficiency are the same patients. The coding captured the cases for both gonadotropin deficiencies separately; therefore, for patients with hypogonadotropic hypogonadism, the physician selected the deficiency for both (a further 12 patients had gonadotropin deficiency and a further 22 patients had secondary hypogonadism). Ten patients discontinued treatment before the end of the study; however, none of the discontinuations were related to the primary or secondary endpoints: four were due to adverse events, one withdrew consent to participate, three for protocol non-compliance; one for protocol deviation; and one owing to high IGF-I levels. The mean (SD) duration of exposure to Saizen^®^ was 248.9 (68.25) days (median 273.0 days; interquartile range 267.0–277.0) and the mean (SD) total dose received per patient throughout the study was 65.0 (31.85) mg (median 63.4 mg; interquartile range 44.2–80.3). Owing to adverse events, missed doses or as part of the titration process, 76 (97.4%) patients required dose adjustment during treatment.

**Table 1:** Patient demographics

Demographics	Patients (*N* = 78)
Sex	Men *n* = 48 (61.5%) Women* n* = 30 (38.5%)
Mean age (SD), years	44.5 (12.6)
Median height (Q1:Q3), cm	172 (164.0:181.0)
Median weight (Q1:Q3), kg	83.5 (72.0:98.7)
Median BMI (Q1:Q3), kg/m^2^	28.1 (25.3:32.1)
Number of GH-treatment naïve patients (%)	64 (82.1%)
Number of GH-treatment-experienced patients (%)	14 (17.9%)
Median time since diagnosis (Q1:Q3), years	3.67 (0.52:11.86)
Number with acquired GHD (%)	71 (91%)
Number with idiopathic GHD (%)	7 (9%)
Mean overall adherence (SD),	89.3% (13.35)
Proportion with adherence > 80%	84.6%
Patients reporting ≥ 1 medical condition related to GHD	72 (92.3%)
Endocrine disorders	80 (89.7%)
Endocrine disorders reported by ≥ 10%	
Adrenocorticotropic hormone deficiency	44 (56.4%)
Diabetes insipidus	23 (29.5%)
Follicle-stimulating hormone deficiency	22 (28.2%)
Gonadotrophin deficiency	12 (15.4%)
Hypothyroidism	9 (11.5%)
Luteinizing hormone deficiency	22 (28.2%)
Secondary hypogonadism	22 (28.2%)

Demographics are described in the safety population (i.e. those patients who had at least one dose of Saizen^®^)

*BMI* body mass index, *GH* growth hormone, *GHD* growth hormone deficiency

### Primary endpoint

No patients in the mITT population presented with BAbs at any time during the trial (Clopper–Pearson 95% confidence interval 0.00–4.68%); this result was confirmed in a per-protocol (sensitivity) analysis (Clopper–Pearson 95% confidence interval 0.00–6.38%).

### Secondary endpoints

**Fig. 2:**
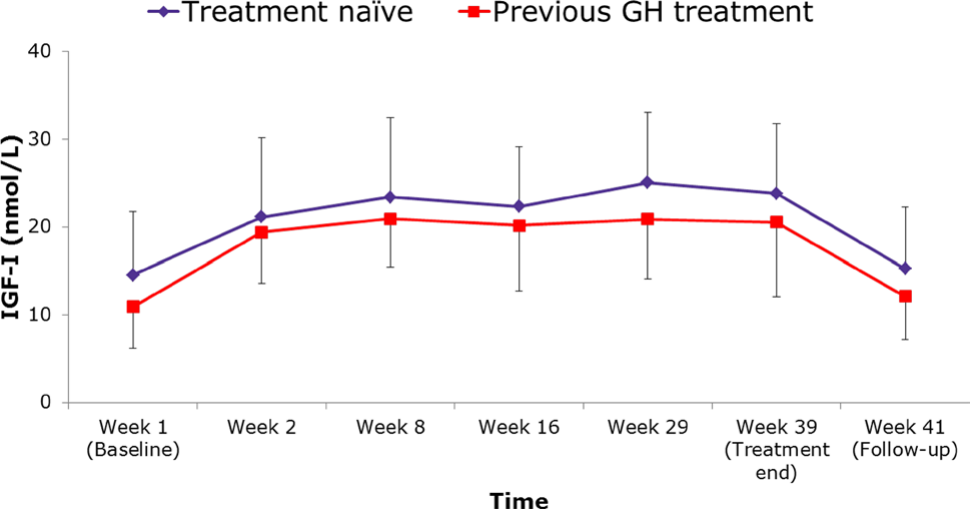
IGF-I concentrations throughout the study period. *GH* growth hormone, *IGF-I* insulin-like growth factor 1

**Fig. 3:**
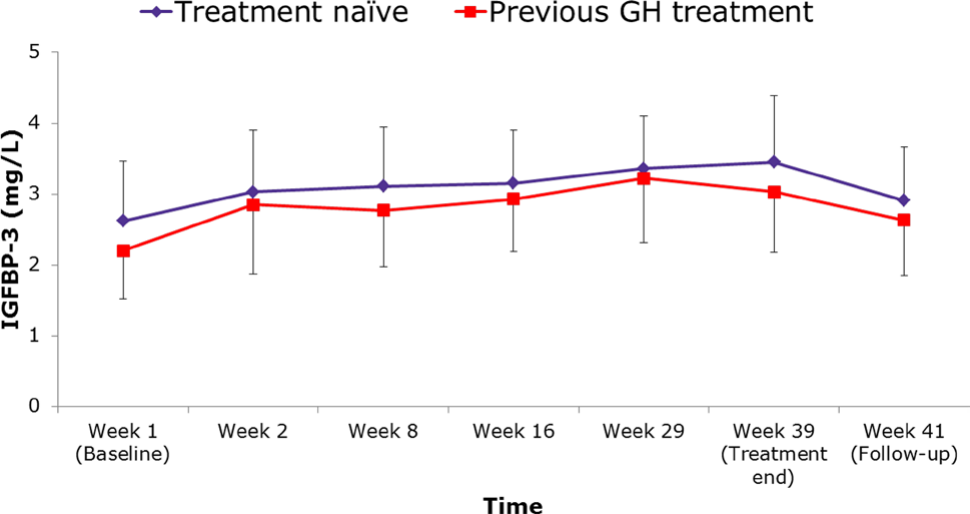
IGFBP-3 concentrations throughout the study period. *GH* growth hormone, *IGFBP-3* insulin-like growth factor binding protein 3

Because no BAbs were detected, no analysis for NAbs was performed. Responses to treatment with Saizen^®^ solution for injection were demonstrated by the changes in IGF-I and IGFBP-3 concentrations, which increased after starting treatment with Saizen^®^ liquid formulation and returned to baseline after treatment was stopped (Figs. 2, 3). Mean IGF-I SDS scores increased slightly (range 0.16–0.23 change in absolute value) during treatment (Table 2). Throughout the treatment period, IGF-I, IGFBP-3 and IGF-I SDS values were higher in the treatment naïve group than in the group previously treated with r-hGH.

**Table 2:** Growth hormone response biomarkers

	Visit 2 (day 1)	Visit 3 (week 2)	Visit 4 (week 8)	Visit 5 (week 16)	Visit 6 (week 29)	Visit 7 (end of treatment)	Visit 8 (follow-up)
IGF-I SDS							
Mean (SD)—treatment-naïve patients	− 3.27 (0.82)	− 3.10 (0.78)	− 3.05 (0.77)	− 3.09 (0.80)	− 3.01 (0.77)	− 3.02 (0.78)	− 3.23 (0.83)
*n* (missing)	64 (14)	64 (14)	63 (15)	59 (19)	59 (19)	61 (17)	56 (22)
Mean (SD) change from baseline—treatment-naïve patients	–	0.16 (0.13)	0.22 (0.15)	0.18 (0.12)	0.25 (0.19)	0.22 (0.17)	0.02 (0.09)
Mean (SD)—treatment-experienced patients	− 3.67 (1.22)	− 3.46 (1.14)	− 3.16 (0.70)	− 3.17 (0.65)	− 3.17 (0.71)	− 3.44 (1.29)	− 3.31 (0.69)
*n* (missing)	14 (64)	14 (64)	13 (65)	13 (65)	13 (65)	14 (64)	12 (66)
Mean (SD) change from baseline—treatment-experienced patients	–	0.21 (0.15)	0.23 (0.16)	0.23 (0.21)	0.23 (0.14)	0.23 (0.20)	0.03 (0.08)

Measured in the safety population (i.e. patients who had at least one dose of Saizen^®^)

*Visit 2* baseline visit, *IGF-I* insulin-like growth factor 1

Mean (SD; range) treatment adherence (proportion of injections received vs. injections prescribed) downloaded from the easypod™ electronic injection device was 89.3% (13.35; 22–102). 12 (15.4%) patients had < 80% adherence, 35 (44.9%) had ≥ 80 to < 95% adherence and 31 (39.7%) had ≥ 95% adherence.

Overall, 72 patients (92.3%) had at least one adverse event during study treatment (Table 3). Most adverse events were either mild or moderate in severity and thought to be unrelated to study treatment. The most common adverse events were infections or infestations [45 patients (57.7%)], nervous system disorders [36 patients (46.2%)], general disorders and administration-site conditions [26 patients (33.3%)], musculoskeletal and connective tissue disorders [25 patients (32.1%)], gastrointestinal disorders [20 patients (25.6%)], and skin and subcutaneous tissue disorders [16 patients (20.5%)]. The most frequently reported adverse events were headache [27 patients (34.6%)], nasopharyngitis [21 patients (26.9%)], arthralgia [13 patients (16.7%)], and back pain [10 patients (12.8%)].

**Table 3:** Adverse events reported during treatment

	Patients (*N* = 78)
Any adverse event	72 (92.3%)
Any severe adverse event	18 (23.1%)
Any serious adverse event	4 (5.1%)
Any adverse event related to the study treatment	21 (26.9%)
Any serious adverse event related to the study treatment	0
Any adverse event leading to study treatment discontinuation	4 (5.1%)
Any adverse event leading to study termination	3 (3.8%)
Any adverse event leading to death	0

Data are *n* (%). Adverse events are described in the safety population (i.e. those patients who had at least one dose of Saizen^®^)

Twenty-one patients (26.9%) reported at least one adverse event that was likely to be related to study treatment; these occurred most commonly in the category of general disorders and administration-site conditions [16 patients (20.5%)]: injection-site bruising [7 patients (9%)], peripheral swelling [3 patients (3.8%)], injection-site pain [2 patients (2.6%)], peripheral oedema [2 patients (2.6%)], chest discomfort [1 patient (1.3%)], injection-site cyst [1 patient (1.3%)], injection-site erythema [1 patient (1.3%)], injection-site haemorrhage [1 patient (1.3%)], injection-site paraesthesia [1 patient (1.3%)], injection-site pruritus [1 patient (1.3%)], and pyrexia [1 patient (1.3%)]. Fatigue and headache [one patient each (1.3%)] were the only severe treatment-related adverse events.

Five serious adverse events were reported by four patients (one patient had an infection, one patient had pneumonia, one patient had esophagitis and iron deficiency anaemia, and one patient had non-cardiac chest pain), all of which were deemed to be unrelated to study treatment. No serious adverse event was related to the study drug (Table 3).

Four patients discontinued the study treatment owing to adverse events (one had a pituitary cyst, one had pneumonia, one had peripheral oedema and one had peripheral swelling): the peripheral oedema and peripheral swelling were considered to be related to study treatment.

All laboratory test results were consistent with the known safety profile for Saizen^®^.

## Discussion

The results reported here confirm the low immunogenic potential of the new liquid formulation of Saizen^®^ and provide further evidence for the efficacy and safety of this formulation. The new liquid formulation will benefit users by removing the need to reconstitute freeze-dried powder, and thus simplify the injection process and provide greater confidence for patients that the correct dose has been administered.

The development of anti-drug antibodies, which can affect efficacy and safety, is an important concern for all biological therapeutics. It is well known that the administration of therapeutic proteins (also called biologics or biotherapeutics) often leads to the induction of antibodies, which can either be neutralising or non-neutralising. NAbs bind to the active site of a therapeutic protein and directly inhibit (neutralise) the therapeutic effect of the product, thereby reducing efficacy. Non-neutralising antibodies (BAbs) bind to antigenic sites in the therapeutic protein without affecting the target binding site; however, they can influence the pharmacodynamics or pharmacokinetics, and will eventually compromise efficacy. The development of such antibodies is usually exposure dependent, and the risk increases in line with several factors, such as time on treatment, changes in the drug formulation, alterations to the protein structure and the manufacturing process, variations between batches of the drug, the route of administration, the dose level and the frequency of dosing [13]. Hence, immunogenicity testing and analysis are required for different pharmacological forms.

During r-hGH treatment, a small proportion of patients develop anti-r-hGH antibodies to somatropin. The incidence of anti-r-hGH antibodies during treatment with somatropin varies between 0 and 8%, as reported in different trials [15–17]. The clinical significance of these antibodies is unknown; most of those detected during clinical trials have been of low binding capacity and have not been associated with growth attenuation except in patients with gene deletions [20–22]. A group of authors recently suggested that the anti-r-hGH antibodies and the GH receptor bind to different epitopes on the GH molecule, which may explain the fact that anti-r-h-GH antibodies generally do not influence growth [23].

Immunological data for the freeze-dried formulation of Saizen are derived from multi-centre trials conducted over 15 years ago. The liquid formulation has demonstrated comparability to the freeze-dried formulation based on clinical pharmacology. An appropriate comparison of relevant quality attributes showed that both products are highly similar and are considered comparable. Although the pharmacokinetics of the new liquid formulation of Saizen^®^ are consistent with those known for r-hGH, and no new safety concerns have been reported [18], we carried out this study to assess the long-term immunogenic potential of the new formulation of Saizen^®^ in adults with AGHD, most of whom were r-hGH-naïve, over 9 months.

No BAbs or NAbs were detected at any point during the trial, which is in line with previous data on the freeze-dried formulation of Saizen^®^ and reported data on other r-hGH products [24–26]. The difference in sensitivity between assays used in the early trials (1980s) and the ones available nowadays could make the comparison between old and recent formulations difficult because the original radioimmunoprecipitation assays have now been superseded by assays with greater specificity and sensitivity for antibody detection, such as the ECL assay used in this study. Although the possibility remains of antibody production below the threshold of sensitivity of the assays used, ratification of the previous immunogenicity results using a fully validated ECL assay adds to the body of data on the low immunogenicity of r-hGH preparations for most patients. In practice, it is likely that antibodies against somatropin would arise in a subpopulation of patients with paediatric growth hormone deficiency (PGHD type A): this population does not produce endogenous somatropin and, thus, their immune system might recognise exogenous r-hGH as foreign.

The number of patients completing the trial falls slightly short of those we anticipated but, because no patients developed BAbs during the study, we consider the results from the patients completing the study to be sufficient to substantiate our conclusions. We do acknowledge that some patients who were treated for ≤ 39 weeks may not have had sufficient time to develop an antibody response and the number of patients exposed for 39 weeks was low; therefore, while medically the exposure was sufficient, we cannot exclude the possibility that antibodies will be generated during treatment over longer periods or in a broader patient population, and continuing pharmacovigilance will be needed.

Mean adherence to treatment in this study was high, as indicated by data downloaded directly from the easypod™ electronic injection device, and the median values for treatment duration and dose received are in agreement with patients receiving the full dose of Saizen^®^ during the study. Despite these considerations, the IGF-I SDS results are below those of their healthy peers for the study cohort throughout the trial [27]. Although the starting doses used in this study were in line with clinical practice guidelines for AGHD, [28], several patients had dose increases during the treatment period that were titrated according to IGF-I concentrations in the age-adjusted and gender-adjusted reference ranges. The IGF-I SDS results for the patients reported here were below those reported for their healthy peers, which may indicate that the dosing of Saizen^®^ in general was not fully optimised. However, efficacy for AGHD was not a primary objective of the study, and response to treatment was reflected only through the IGF-I and IGFBP-3 concentrations, which were analysed in the safety population with no imputation for missing data. Furthermore, as these data reflect the situation in clinical practice, decisions about dose adjustment were at the discretion of the treating physicians, and some patients may have been prescribed a dose that was lower than the lowest specified starting dose for the study, even though the mean duration of exposure and mean dose were consistent with patients receiving treatment in accordance with the trial protocol. The outliers in the safety population had values substantially below those anticipated from compliance with the trial protocol: the lowest for adherence was 22%, the lowest duration of treatment was 19 days and the lowest overall dose was 4 mg. Investigation of the reasons for such low compliance at the individual level was, however, outside the scope of this study.

Overall compliance to treatment is comparable to other studies (85.7% in children [28]) with similar duration, although it has been reported that adherence can be different in other populations [24–26] and can even drop below optimum rates. The data reported here reflect real-world data on patient usage, meaning that many factors could have had a bearing on patient and caregiver adherence that could not be predicted from the factors that affect adherence during a clinical study. Several factors are known to influence adherence (e.g. age, adolescence, socioeconomic status and family support, education levels, treatment duration, medication issues and lack of medication effect), and an adult population would be expected to be more compliant with the treatment regimen than younger patients. Despite this, in this trial almost 15% of the safety population had adherence below the acceptable level of 80%. Non-compliance in an adult population, who receive lower doses of Saizen^®^ than children with GHD and are therefore less likely to discontinue because of adverse events [28], may be best explained by well-being during the trial or even the absence of a discernible clinical effect during the course of the study, leading to a lack of motivation to adhere to the treatment regimen, which may have implications for the results of the immunogenic potential.

Adult patients with GHD potentially face long-term treatment with r-hGH, and the efficacy, safety and immunogenic potential of prolonged treatment have still to be fully assessed. Therefore, continued pharmacovigilance will be needed to assess the real-world outcomes of on-going treatment, not only on the immunological response, but also on efficacy, safety and compliance [29].

## Conclusions

The results of this study support the low immunogenic potential of the liquid formulation of Saizen^®^, consistent with the immunogenicity data for the freeze-dried formulation, in an adult population with biomarker-confirmed responses to treatment. The overall safety profile for the liquid formulation is also in line with that for the previous formulation, and no new safety concerns were detected.
